# The Interest of the Dentist *Versus* the Interest of the Public

**Published:** 1904-09

**Authors:** R. Anema


### THE INTEREST OF THE DENTIST *VERSUS* THE INTEREST OF THE PUBLIC.[1]

BY R. ANEMA, D.D.S.

Upon receiving the kind invitation of the Chairman to read a paper before your society, I felt very much pleased, and gladly accepted it, because I know there are among you many men who are more learned and more able than I am to give valuable suggestions upon a subject in which I am very much interested. The subject, to which I hope you will give some thought, is "The Interest of the Dentist *versus* the Interest of the Public." The main feature, which makes the title less arbitrary than you at

---

[1] Read before the thirty-sixth annual session of the Pennsylvania State Dental Society, July 12, 13, 14, 1904.

first might have thought, and of which I am going to present you as a study, is "professional egotism." I say *study* purposely to let you know that my paper, instead of being a well-designed and nicely framed picture, is merely a sketch.

Problems such as we have to consider here may be called in some ways sociological problems. In trying to resolve such questions, it seems next to impossible to leave out the mental factors called moralizing and preaching, as well as the fiercer one of condemning. These factors are dangerous friends when we earnestly wish to probe a sociological question to its bottom. In endeavoring to come as near to the truth as possible, let us therefore leave those three passionate friends at home and look upon things as the German philosopher, Nietzsche, would do, from a point on the other side of good and evil.

I am unable to give you a synthesis of professional egotism, by which you might see at once its whole spiritual body, with its clear outlines and distinct limits. The method I shall follow is the one of analysis and the study of symptoms as I see them or mean to see them.

Perhaps it is unnecessary to say that this way of treating the subject will occasionally show a hiatus on the road on which the essayist leads you, or, in other words, will occasionally give the essay a somewhat incoherent character.

In my language, the Netherland language, there are two words for your one word "profession," both words being almost equally common in use. The one gives the concrete signification of profession, meaning the number of class of men that practise the same art; the other gives the abstract signification, and means vocation or calling. With these two words in mind, it is easier to make one's self understood than with one. The concrete word class for dental profession is a rather unfamiliar word in American society, because class differences are not as distinct as in older countries, and perhaps, also, because the atmosphere in the younger world is oftener and more heavily attacked by idealistic storms. When one keeps in mind only the abstract signification of profession, professional egotism is a *contradictio in terminis,* but when one thinks of the concrete the new term becomes more familiar.

To my mind there is no more doubt of the existence of professional egotism that there is of a certain kind of patriotism, called jingoism, that says, "My country, right or wrong," or of that egotism which, by instinct and brute force, extinguishes weaker races. Of the latter, we have in the history of mankind many instances. Of the former, I hope to give you an instance later on. All these forms of egotism are brought about by instincts of the individual becoming active in the mass at certain times and intervals of its existence. In seeking the genesis of professional egotism, allow me to tell you first something of the instinct of self-preservation, and then of what I call self-preservation of the class.

In our days when education and civilization are overestimated, perhaps it needs some courage to declare that even a dentist is an ordinary human being, with instincts giving active power to his mind and body.

One of your best authors says that "Of all animals, man has the most instincts." Among the strongest instincts of man is the one of self-preservation. It is a common truth that instincts are hard to deal with, if, indeed, they can be dealt with at all. They may become concealed to the inexperienced eye, covered by so-called civilization, which oftentimes is nothing but a social veneer, or by an amiable self-deceit of good people, which makes them believe that at least among the higher classes instincts, and especially the one of self-preservation, are rudimentary. If, however, these good people believe that those higher classes look out merely for the interests of others, they are mistaken. The lesson given nearly two thousand years ago, "Love thy neighbor as thou lovest thyself," is still generally applicable, which means that man, as a rule, loves himself more than his neighbor and more than any other man. His instinct of self-preservation dominates his altruistic feelings. I will not say, however, that there are no such people as martyrs, in which this instinct seems almost lost, but this specimen is very rare.

When a man joins a profession he brings his instinct of self-preservation with him. It brings him quite often in closer contact with the profession, as he hopes that the profession may at some time be of some use to him. Notwithstanding man's idealistic feelings and the almost overwhelming influence of them, as is the case after long solitary contemplation, or transferring thoughts to others in enthusiastic gatherings, it cannot be denied that the average professional man not only possesses this instinct of self-preservation, but that he needs it wherewith to earn his daily bread.

When we take the dental profession as a class, each man making a living through dentistry, to protect himself and his family from starvation, should it be at all surprising that there exists such a thing as self-preservation in this class? Again, our professional family may be compared with the still larger family of nation or race, in which the instinct of self-preservation is always present, being especially awake and active in times of rude competition, called war, but dormant at times where no attacks are to be feared. The following, I think, is an example of professional self-preservation: In a certain country a society of dentists, forming the editorial staff of a dental journal, desired to change the rules. One of the members proposed the following as the principle on which the new structure should be erected:

"The society intends to serve the public by promoting dentistry, dentistry in the most remote sense of Art and Science." His obvious reason, as he explained at the time, was to have as the leading motive the "interest of the public." After having been carefully considered, the proposition was rejected almost unanimously. So far as the thoughts of the opposition could be understood, the men who voted against the "public interest" proposition, feeling themselves representatives of their profession, considered it their duty not to look out—at least not in the first place—for interests other than those of the body of men represented. The new rules were now based upon a foundation which can easily be laid bare and understood by looking upon the flag and emblem of one of our best dental journals, which says "Devoted to the interests of the profession."

As dental journals are leaders and at the same time the voice of the profession, I consider these two facts as very valuable ones and as a proof that the dental profession needs devotion to its own interests; and as devotion to one (the profession) excludes devotion to another (the public),—otherwise it is no devotion,—what else can be the cause for the demand of this highest form of affection than the instinctive self-preservation of the class? But as an instinct acts without consciousness of the purpose, its purposive action may not be generally observed, and therefore my conclusion that we have with these two journals also two instances of self-preservation of the class may not be generally accepted.

Judging from these cases and comparing them with similar ones in other countries, I think I am right in saying that the dental profession, as all young bodies, has a strong instinct of self-preservation, and at the same time is limited in its motive power, handicapped as it is on its way to the general ideal, "the interests of the public," by its class instinct, "the interest of the profession."

After this I may give you at once my definition of professional egotism as the *aggressive self-preservation of the class.* This potent inner prompting becomes fully awake and highly active in times and places where attacks are to be feared by competition, in the same way as wars are brought about by collision of the interests of nations.

Here follows an instance of professional egotism: In a certain country a body of dentists use their power to prosecute respectable foreign dentists for using legally acquired but foreign degrees of doctor. As their country does not furnish a dental doctor degree, the dentists claimed that the foreigners gained an undue reputation by the use of that title, thus doing harm to the interests of the local dentists.

Here I might have finished my paper on the "interest of the dentist *versus* the interest of the public and professional egotism," but as I feel somewhat in debt to the original title, especially to the last part, "the interest of the public," I hope you will allow me to make a few more remarks.

If it is true that the profession as a body is limited in its motive power (being devoted to its own interests), an interesting question to treat, especially from the public point of view, is, "Who looks out for the interests of the public in the case of dental legislation?" Dental legislation, according to some, exists to protect the public from malpractice, and, according to the same, is necessary for that purpose.

However, it cannot be according to this principle that a law is made which debars a foreigner from practising his art, no matter from which reputable school he comes or how many years of reputable practice he may have had, but compels him to pass through all the preliminary examinations and to spend at least two years in the local dental school, after which he must pass the dental examinations. And yet there is such a law in France.

And what shall we think of the legislation of many of the American States, as cited by Dr. Kirk in his paper on "The Unification of Dental Legislation," when he says, "Much of our dental legislation owes its existence to the effort of those who make the protection of the dental practitioners against the competition of his unqualified neighbor his primary and leading motive"?

And to think of the professional legislation of Austria, Belgium, and Italy where only medical men are allowed to practise dentistry, and that, too, without the necessity of passing any dental examination at all!

And when all the European countries give to their physicians a right to practise dentistry, do you think that all this legislation is made in the interest of the public, or merely to satisfy a class that at the time has the most influence upon the legislature? The leading motive in all these cases may have been protection of the public from malpractice, but if it was, in my opinion the object has not been gained.

It may surprise some of you that in Germany the healing art is free. Every one who likes may practise medicine and also dentistry. It has been a long time an open question to me why this should be, but in looking back upon professional legislation, perhaps we may come to the conclusion that Germany is not so wrong after all. Anyway, we know that Germans are a philosophical race, and think a long time before they act. Is it merely accidental that in Germany the philosophical thought originated that the main desire in man was the desire for power?

These were the suggestions I owed to the "interests of the public."

At the end I apologize for what moralizing I have done. Notwithstanding I have endeavored to hold back my friends, the preacher and the condemner, I know that I have not fully succeeded.

Now, when I am free again, I hope you will allow me a few moralizing remarks. That I have not spoken more for "the interest of the dentist" I hope will not be a reason for accusing me of criminal neglect, as there is sufficient proof that the dentist can look out for his own interests. Still, I fear that some of you will condemn me for talking so much of professional egotism and for exposing the profession as I have done, and, as I dislike to be censured, I promise that, at some future time, I will write a still longer essay on "professional altruism," in that way turning my critics into friends. Because, with Plato, I am a firm believer in the absolute good, a spirit which is also largely represented in our profession. However, the sun does not always shine, neither does the good.

